# First evidence of the mutations associated with pyrethroid resistance in head lice (Phthiraptera: Pediculidae) from Honduras

**DOI:** 10.1186/s13071-020-04183-2

**Published:** 2020-06-16

**Authors:** Kelsey Larkin, Carol A. Rodriguez, Shabana Jamani, Georgina Fronza, Gonzalo Roca-Acevedo, Ana Sanchez, Ariel C. Toloza

**Affiliations:** 1 Department of Health Sciences, Faculty of Applied Health Sciences, Brock University, St. Catharines, ON Canada; 2 School of Microbiology, National Autonomous University of Honduras (UNAH), Tegucigalpa, Honduras; 3 Instituto de Investigaciones en Microbiología, Universidad Nacional Autónoma de Honduras, Tegucigalpa, Honduras; 4 Centro de Investigaciones de Plagas e Insecticidas (UNIDEF-CONICET), Villa Martelli, Argentina

**Keywords:** Pediculus humanus capitis, Insecticide resistance, kdr, Voltage-sensitive sodium channel, Pyrethroid, Honduras

## Abstract

**Background:**

The human head louse, *Pediculus humanus capitis*, is a cosmopolitan blood-sucking ectoparasite affecting mostly schoolchildren in both developed and developing countries. In Honduras, chemical pediculicides are the first line of treatment, with permethrin as their main active ingredient. Despite the extended use of these products, there is currently no research investigating insecticide resistance in Honduran head lice. In head lice, the most common mechanism is knockdown resistance (*kdr*), which is the result of two point mutations and the associated amino acid substitutions, T917I and L920F, within the voltage-sensitive sodium channel (VSSC).

**Methods:**

Genomic DNA was extracted from 83 head lice collected in the localities of San Buenaventura and La Hicaca, Honduras. Polymerase chain reaction (PCR) was used to amplify a 332-bp fragment of the VSSC gene that contains a site affected by C/T mutation which results in a T917I amino acid substitution on each human head louse genomic DNA fragments.

**Results:**

The C/T non-synonymous mutation which results in the T917I *kdr* amino acid substitution was detected in both head lice populations at frequencies ranging between 0.45–0.5. Globally, the frequency of this substitution was 0.47. Of these, 5 (6.1%) were homozygous susceptible and 78 (93.9%) were heterozygotes. The *kdr*-resistant homozygote (RR) was not detected in the studied populations. Thus, 93.9% of the head lice collected in Honduras harbored only one T917I allele. Exact test for the Hardy-Weinberg equilibrium for both localities showed that genotype frequencies differed significantly from expectation. In addition, San Buenaventura and La Hicaca populations had an inbreeding coefficient (F_is_) < 0, suggesting an excess of heterozygotes.

**Conclusions:**

To our knowledge, this is the first study showing the presence of the C/T mutation responsible of the T917I kdr allele associated with pyrethroid resistance in *P. h. capitis* from Honduras. The PCR-restriction fragment length polymorphism (RFLP) employed here has demonstrated to be a reliable, economic, and reproducible assay that can be used to accurately genotype individual head lice for the mutation encoding the resistance-conferring T917I amino acid substitution. This highlights the necessity of proactive resistance management programmes designed to detect pyrethroid mutations before they become established within populations of head lice.
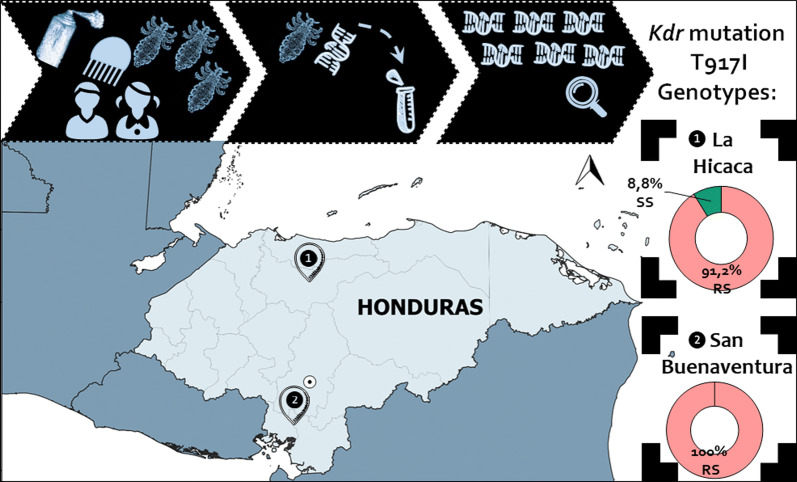

## Background

*Pediculus humanus capitis* De Geer, commonly known as the human head louse, is a cosmopolitan and obligate ectoparasite found both in developed and developing countries. Head lice infestation is known as pediculosis capitis which can result in pruritus, loss of sleep, attention deficit, and is associated with secondary skin infections due to scratching of an irritated scalp [1]. Head lice are transmitted by direct host-to-host contact, and are heavily influenced by both social and behavioral transmission factors in children 4–13 years-old [2]. Despite the fact that head lice do not possess a disease vector status, they have been found to carry bacteria associated with typhus (only under controlled experimental conditions), trench-fever, and hospital acquired infections [3–6].

The control of pediculosis among populations comprises both individual and collective approaches. The former includes the use of mechanical or chemical methods, while the latter is based on limiting exposure through behavioral modifications. Specific treatment for this infestation relies heavily on the topical use of pediculicides, compounds with toxic or otherwise deleterious effects to lice. Traditionally, pediculicides containing a wide variety of insecticides such as DDT, lindane, carbaryl, malathion, *d*-phenothrin, and permethrin have been employed to treat head lice infestations worldwide [7]. Chemical pediculicides are still recommended as first line of treatment worldwide and consist of over-the-counter (OTC) products, as well as products only available under medical prescription.

With sales over 200 million USD per year in the USA market alone, the global industry for topical OTC products play a significant role in the control of this infestation [8]. The most frequently used pediculicides are topical formulations, notably those containing pyrethroids and organophosphates as their main active ingredients. Of these, pyrethroids are the most widely used due to the shorter exposure time, less odor, lower mammalian toxicity, and relatively safe environmental persistence. However, the intensive and continuous use of this class of insecticide has led to the development of resistance, ultimately hindering control strategies in several countries like Denmark, UK, France, Israel, Argentina, USA, México, Russia and Chile [4, 9–16].

Pyrethroid resistance is perhaps the single most important factor for the increased prevalence of head lice infestations worldwide [17, 18]. Lice resistant to pyrethroids possess “knockdown” resistance (*kdr*), caused by single nucleotide point mutations (SNPs) in the *para*-orthologous voltage-sensitive sodium channel (VSSC) gene, which result in reducing nerve sensitivity to the insects. It has been clearly established that the primary amino acid substitution, T917I and L920F, located in domain II, are responsible for resistance [19]. A number of other mutations have been reported, but they are not good candidates as molecular markers of resistance. The mutation D11E is unlikely to be involved in insensitivity of the sodium channel, as it is conservative, located in an N-terminal inner membrane segment, and found in susceptible body lice [20]. Mutations T929I and L932F, which have been reported as associated with permethrin resistance were in fact expressed in the amino acid sequence positions of the house fly VSSC (rather than in the head louse amino acid sequence). Moreover, it has been demonstrated that these mutations coexist *en bloc* as a resistant haplotype, and when T197I was expressed in *Xenopus* oocytes either alone or in combination, virtually suppressed permethrin sensitivity. A body of evidence points to the relevance of T917I amino acid substitution in pyrethroid resistance *via* the *kdr*-type nerve insensitivity mechanism, and therefore can be used as a molecular marker for resistance detection [21].

In Honduras, a few authors have reported that pediculosis is a neglected and serious health problem affecting 10–83% of school-aged children from several regions [22–24]. Chemical lice control in Honduras is ubiquitous, with an abundance of pyrethrin and pyrethroid-based products in the market (e.g. pyrethrin 1%, permethrin 1–4%, and cypermethrin 0.2%). Notwithstanding, there is a paucity of research on head lice, and no data about head lice insecticide resistance are available for Honduras. With the exception of anecdotal reports about the inefficacy of OTC pediculicides [24], whether insecticide resistance is emerging among head lice populations in the country remains unknown. The present study aimed to investigate the presence and distribution of the non-synonymous mutation responsible of the T917I *kdr* substitution in head lice from two localities of Honduras.

## Methods

### Lice samples

**Fig. 1:**
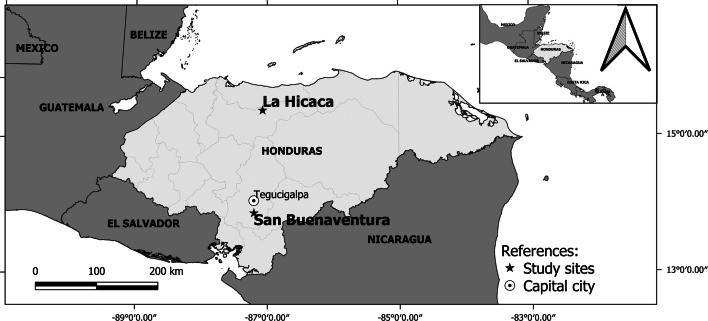
Geographical location of the head louse populations from Honduras

A total of 83 human head lice (adults and stage III nymphs) were collected from children residing in two Honduran rural communities (La Hicaca and San Buenaventura) (Fig. 1). Lice were collected from heads of infested children using a dry-combing technique with the aid of an electric V-comb (ToLife Technologies, Welshpool, Australia), a stainless-steel metal comb that utilizes suction power to trap head lice and nits into single-use filters [24]. An adjustable headset with magnifying lenses (MG81001-G-2 led Headband illuminating Magnifier 2; ToLife Technologies) to inspect the head in order to identify the different insect stages was used. Stainless steel tweezers were used to collect live lice that were trapped in the head of the V-comb. Overall inspection of the whole head was around 20–30 min, depending on hair length and thickness. Between inspections, the head of the V-comb, hair clips, and tweezers were sanitized in 10% bleach solution and rinsed with clean water. Then, lice were transferred from the filters into vials containing 70% ethanol. Once in the laboratory, insects were stored at 4 °C until studied

### Extraction of genomic DNA and PCR amplification of the *kdr*-fragment

Genomic DNA was isolated from individual adults and nymphal stage III head lice following the methodology of Ascunce et al. [25]. In short, each louse was cut in half using a scalpel, placed in a 1.5 ml Eppendorf tube containing cell lysis solution and proteinase K, then homogenized using a sterile plastic pestle. DNA was extracted from each individual louse using the Wizard genomic DNA purification kit (Promega, Madison, WI, USA) following the manufacturer’s protocol for insect tissues. After extracting the DNA, a dilution from the original concentration to ≈5–10 ng/µl was made.

The RFLP method employed here uses genomic DNA to discriminate between resistant and susceptible VSSC gene alleles in head lice and as described by Kristensen et al. [9]. This method allows for easy discrimination of three genotypes (homozygous susceptible, heterozygous and homozygous resistant) by the number and size of electrophoretic bands detected in an agarose gel. Other advantages of this method include cost-effectiveness, efficiency and reliability. Most importantly, this method allows zygosity detection, which is particularly relevant in epidemiological studies [11].

Polymerase chain reaction (PCR) was used to amplify a 332-bp fragment of the VSSC gene that contains a site affected by a C/T mutation corresponding to the T917I amino acid substitution on each human head louse genomic DNA fragments [11].

Reactions consisted of a total volume of 25 µl including 12.5 µl MasterMix (Promega), 1 µl (0.25 µM) of each primer (sense: 5’-AAT CGT GGC CAA CGT TAA A-3’; antisense: 5’-TGA ATC CAT TCA CCG CAT AA-3’), 2 µl of total genomic DNA, and 8.5 µl of ddH_2_O [13]. The PCR reactions were programmed as follows: 94 °C for 10 min; 40 cycles of 94 °C for 30 s, 56 °C for 30 s, 65 °C for 1 min; and a final extension step at 65 °C for 10 min. Then, 10 µl of each PCR amplicon was digested with 10 U of *Ssp*I restriction enzyme (Thermo Fisher Scientific, Waltham, USA) at 37 °C to determine the AAT^˄^ATT restriction site. Digested fragments were separated using 2% agarose gel electrophoresis, visualized by staining with ethidium bromide, and analyzed after being photographed under UV light.

### Statistical analysis of genotype frequencies

Genotype frequencies were calculated by dividing the number of lice of each genotype (RR, RS and SS) by the total number of analyzed head lice. Then, genotype frequencies at the 917 locus were tested to fit the Hardy-Weinberg (H-W) expectations using the program Genepop (v. 4.2) [26], option 1 (Hardy-Weinberg exact tests), sub-option 3 (probability test; [26]). This software was also used to estimate Wright’s inbreeding coefficient (F_is_) [26] and for populations out of the H-W equilibrium. These values were employed to test for heterozygote deficiency and excess (Genepop option 1, sub-options 1 and 2, respectively) using the U test as described in Raymond et al. [27].

## Results

We identified and determined the presence of the T917I *kdr* substitution in all 83 head lice collected (Table 1). The diagnostic genetic marker linked to permethrin resistance consists of the presence of one or two fragments after the digestion with *Ssp*I. The occurrence of the nucleotide substitution of the C→T that codes for the T917I substitution results in a unique cutting site of this restriction endonuclease in the *kdr*-fragment. Thus, three possible amplified fragments leading to three head louse genotypes were found: a homozygous susceptible or wild-type allele- (SS) identified by a fragment of 332 bp; the heterozygote (RS) with three fragments of 332, 261 and 71 bp; and a homozygous resistant mutant allele-identified by two bands of 261 and 71 bp. Globally, the frequency of the T917I substitution was 0.47 in the two head louse populations (La Hicaca and San Buenaventura) of Honduras. Both lice populations possessed *kdr*-like alleles with a medium frequency of 45.6 and 50%, respectively. Of these, 5 (6.1%) were homozygous susceptible and 78 (93.9%) were heterozygotes. The *kdr*-resistant homozygote (RR) was not detected in the studied populations. Thus, 93.9% of the head lice collected in Honduras harbored only one T917I mutant allele.

**Table 1:** Distribution of *kdr*-like alleles T917I in head lice populations from Honduras

Population	No. of head lice analyzed (no. of infested subjects)	Genotype^a^			Resistance allele frequency (%)	H-W^b^ (*χ*^2^)	F_is_^d^
		S/S	R/S	R/R			
San Buenaventura	25 (8)	0	25 (100)	0	50	25^c^	− 1^c^
La Hicaca	58 (12)	5 (8.6)	53 (91.4)	0	45.68	41.04^c^	− 0.83^c^
Total	83 (20)	5 (6.1)	78 (93.9)	0	46.98	65.2^c^	− 0.887^c^

^a^S and R are susceptible and resistant alleles. Between brackets are the percentages of each genotype proportion

^b^Populations were tested for the Hardy-Weinberg equilibrium by a chi-square test (*χ*^2^ = 3.84, *df* = 2, *P* < 0.05)

^c^Values that are statistically significant at *P* < 0.05. Significance level indicates rejection of the null hypothesis F_is_ = 0 at *P* < 0.05

^d^F_is_ values > 0 indicate heterozygote deficiency, whereas F_is_ values < 0 indicate heterozygote excess

The exact test for the Hardy-Weinberg equilibrium for both localities showed that genotype frequencies differed significantly from expectation. In addition, San Buenaventura and La Hicaca populations had an inbreeding coefficient (F_is_) < 0, suggesting an excess of heterozygotes (Table 1).

## Discussion

Prevalence of head lice infestation varies considerably among populations, with factors such as gender, age, and sociocultural characteristics driving important differences within populations. Despite the label of “neglected tropical disease”, pediculosis is prevalent in both high-income societies as well as resource-poor countries [18]. Control of pediculosis in schools and day care settings are based on health education, early diagnosis, and prompt treatment of infested children. Among the diversity of removal and treatment methods (fine-tooth combing, household products, heat application, oral treatment, and topical insecticides), permethrin remains as the popular chemical compound for the treatment of head lice infestations worldwide. Since the launching of permethrin as an OTC product in the 1980s, its extensive and intensive use for over 30 years has exerted a strong selective pressure on over exposed insect populations across the world [28].

To the best of our knowledge, this is the first study analyzing the permethrin resistance status of *kdr*-type mutations in head lice from Honduras. The frequency of the *kdr* molecular marker was at 0.4698, with an overall mean of 46.95% for the heterozygous lice (RS). These values are in accordance with those found in head lice collected in schoolchildren from Wales and Chile [10, 16]. In these studies, the global percentage of the T917I substitution was 0.43 and 0.50, with 77.2 and 88.8% of the lice characterized as heterozygous; respectively. Despite the similar trend of the *kdr* mutant allele occurrence in the *P. h. capitis* from Wales, Chile and Honduras, the frequency of pyrethroid resistance gene is highly variable and widespread. A world *kdr* map of head louse populations from 14 countries, including North and South America, Asia, the European Union, Oceania, and Africa showed an overall resistance allele frequency ranging between 29–100% [29]. This indicates that geographical variability is highly fluctuating and is exclusively dependent on the selective pressure induced by the use of pyrethroids to treat pediculosis.

In Honduras, research on head lice infestation is scarce, with only three studies available in the literature [22–24]. In these investigations, reported prevalence of head lice varied from 10 to 83%, which is well above the overall 5% epidemiological value considered to be of epidemic importance [30]. As in many countries, pediculosis is a neglected infestation in Honduras which is not prioritized in national or regional public health programmes. In fact, ectoparasitic diseases are included in the strategic plan for the prevention, attention, control, and elimination of neglected infectious diseases in Honduras [24]. Currently, permethrin and cypermethrin are the most commonly used OTC pediculicides in the country, with permethrin being the most available compound with 83% of the market share (CAR, personal communication). As a result, head lice remain exposed to pyrethroid-based pediculicides at a high selective pressure. An exact test for the Hardy-Weinberg equilibrium showed that genotype frequencies differed significantly from expectation in all the studied populations. The deficiency of both susceptible (5 per 83) and resistant homozygous (0 per 83), and the over-representation of heterozygotes (78 per 83), suggests that the head lice populations of Honduras are currently under active selective pressure.

Similarly, the pattern detected in Honduran specimens was also found in head lice from school children of Wales and Chile. For instance, the study performed in Wales showed that of the 316 analyzed lice, 55 were homozygous susceptible, 17 were homozygous resistant and 244 were heterozygotes [10]. In Chile, 5% and 6.1% were both homozygous susceptible and resistant, and 88.9% were heterozygotes [16]. Conversely, head lice collected from France, Argentina, USA and Russia harbored a high frequency of homozygous *kdr*-type mutations suggesting that these alleles are strongly established and almost in fixation [4, 11, 13, 14, 31]. The evolutionary pattern found in the Honduran head lice suggests that if the selective pressure exerted by the pediculicides continue, it is highly probable that the *kdr*-type mutations might increase their frequency and reach fixation.

There are two possible explanations that might help to understand the excess of heterozygotes (93.9%) reported in the present study. The first and the most conservative option is that *kdr*-mutations possess little or no effect on the overall fitness of the individuals, resulting in a slow return of the resistant population to the susceptible state. In other words, the resistant alleles might persist in high frequency within populations [32, 33]. Permethrin resistance *via kdr* mutations was not associated with any fitness disadvantage in head lice from the USA [34]. It could be speculated that head lice have developed means to minimize any fitness disadvantage associated with these resistance mutations. Because the two mutations exist *en block* as a resistant haplotype, the occurrence of the mutation (L920F) may function to compensate for any fitness disadvantage related with the (T917I) amino acid substitution in permethrin-resistant head lice [21]. This suggests that in an environment with no insecticide pressure, lice harboring the *kdr*-like alleles can compete equally with those who lack the mutations (wild type individuals) [35]. The second option to explain the excess of heterozygotes is that there may be a significant fitness cost associated with this genotype. Lice harboring only one copy of the *kdr* mutation may have higher chances to survive under a selective environment compared to lice carrying two copies of the mutation.

A study in Burkina Faso found that heterozygote *Anopheles gambiae* male mosquitoes had a fitness advantage over the homozygote susceptible ones [36]. In that study, heterozygote males had higher mating success than either resistant or susceptible homozygotes, suggesting of a heterozygote advantage effect of the *kdr* mutation in *An. gambiae*. Specifically, this research showed that there is a fitness cost related to possessing double alleles of the 1014F mutation rather than having just one allele. The swarms where the mosquitoes segregated to mate were predominantly composed of homozygote resistant males; however, the heterozygous males were more frequently selected by females for mating. This reduced mating success in homozygote RR *kdr* males, affecting the neural network of the VSSC, consequently impairing some physiological traits such as mobility, perception of stimuli, and detection of olfactory signals. Reduced fitness of homozygous RR *kdr* males would play a significant role in slowing down the expansion of resistance allele L1014F in the wild mosquito populations [37].

Considering that permethrin resistance is mainly mediated by *kdr* mutations, and is determined by the intensity and frequency of the control measures employed to control pediculosis, different strategies could be implemented. In head lice populations where resistance is low or near zero, pyrethroids should continue to be used in conjunction (if possible) with resistance-monitoring programmes. On the contrary, in populations with high resistance levels, pyrethroids should be discontinued and replaced by products with different modes of action. Finally, in populations with intermediate allele frequency, as is the case in Honduras, it is imperative to implement insecticide-resistance programmes through the monitoring and prevention of head lice infestations.

For the establishment of an adequate resistance management programme, it is essential to detect early levels of insecticide resistance while at the same time preventive measures to avoid its spread are promoted. Early resistance detection by traditional toxicological bioassay-monitoring methods is highly recommended but impractical and difficult to operationalize. To overcome these limitations, the PCR-restriction fragment length polymorphism (RFLP) employed in the present work has demonstrated to be a reliable, economic, reproducible assay that can be used to accurately genotype individual head lice for the resistance-conferring T917I mutation.

The findings presented here support the need for more basic and implementation research in Honduras. Of crucial importance is the creation of regional health surveillance programmes focusing on head lice and their control.

Finally, there is an urgent need to increase awareness among the population and health care providers of the dangers of using harmful products and products not labeled to treat human pediculosis. Implementing this recommendation would considerably eliminate the unnecessary overexposure of children to pesticides in order to reduce both acute and chronic intoxications.

## Conclusions

To the best of our knowledge, the findings of this work show for the first time that the T917I *kdr* substitution was detected in two head lice populations from Honduras, with an overall frequency of 0.47. Distribution of *kdr* genotypes differed significantly from Hardy-Weinberg proportions. Thus, deficiency of susceptible (6.1%), lack of resistant (0%) homozygous, and the overrepresentation of heterozygotes (93.9%) suggests that the studied populations of head lice of Honduras are currently under active selective pressure of pyrethroids. This highlights the need for proactive resistance management programmes designed to detect pyrethroid mutations before they become established within populations of head lice.

## Data Availability

Data supporting the conclusions of this article are included within the article. The datasets used and analyzed during the present study are available from the corresponding author upon reasonable request.

## Abbreviations

*kdr*: knockdown resistance
PCR: polymerase chain reaction
RFLP: restriction fragment length polymorphism
VSSC: voltage-sodium sensitive channel
OTC: over-the-counter
SNP: single nucleotide polymorphism
